# Artificial Intelligence—Assisted Monitoring of Water Usage for Cooling Cows on a Dairy Farm

**DOI:** 10.3390/ani15233470

**Published:** 2025-12-02

**Authors:** Fernando Valle, Kelly Anklam, Dörte Döpfer

**Affiliations:** Department of Medical Sciences, School of Veterinary Medicine, University of Wisconsin-Madison, Madison, WI 53706, USA; kelly.anklam@wisc.edu (K.A.); dopfer@wisc.edu (D.D.)

**Keywords:** artificial intelligence, water management, dairy farming, cooling

## Abstract

High-yielding lactating cows generate considerable internal heat, making thermoregulation challenging in warm conditions. Traditionally, sprinkler systems have cooled dairy cows by spraying water droplets onto their skin to aid heat dissipation, especially when used with fans. This study explores the benefits of AI-assisted monitoring of water usage for cooling dairy cows, aiming to optimize water consumption and enhance sustainability. An object detection model, trained with 200 random images from a fisheye security camera installed above pens of dairy cows in a dairy farm, was used to detect the presence or absence of cows in headgate sections to guide water sprinkler activity. According to the object detection model, the implementation of AI-assisted detection of cows’ presence or absence in headgates with an accuracy of 0.924 has the potential to save up to 75 percent of water annually for cooling cows. Additionally, the model can detect cows’ behavior patterns regarding location in the pens depending on the occurrence of heat stress. The implementation of AI-powered detection systems in dairy farms has been proven to enhance sustainability and significantly reduce expenses by curbing the excessive use of water.

## Simple Summary

Water is a vital resource that plays a crucial role in agriculture, especially in dairy farming. This study aims to improve how we keep dairy cows cool and comfortable, which is essential for their health. Many traditional cooling methods rely on set schedules, but these may not always meet the cows’ needs at different times of the day. Instead, we suggest using advanced technology to observe how cows react to their environment in real-time. By monitoring when cows go to cooler areas within their barn or pasture, we can create a smarter cooling system that turns on when the cows really need it. This approach not only prioritizes the comfort of the cows—helping them avoid heat stress and related health problems—but also helps save water using only what is necessary for cooling. By being more efficient with our water use, we aim to promote a more sustainable way of dairy farming that benefits both animals and the environment.

## 1. Introduction

Given the rise in global temperature and the multiple factors associated with global warming, water is used to cool and improve the living conditions of dairy cows [1]. To ensure cow welfare, it is imperative to prevent heat stress, which is achieved using water cooling, shade, and adapted housing systems [2].

Dairy cows, particularly high-yielding lactating ones, generate significant internal heat, which poses a challenge for thermoregulation, especially under conditions of elevated ambient temperature, humidity, or heat stress. When subjected to these stressors, physiological responses include an increase in body temperature, heightened respiratory rates characterized by panting, reduced feed intake, diminished milk production, compromised fertility, suppressed immune function, and exacerbated welfare concerns [3].

To mitigate heat stress, implementing evaporative cooling techniques proves effective. This involves applying water to the skin and hair coat, followed by an airflow mechanism to facilitate evaporation. This process not only lowers the skin and coat temperature but also decreases core body temperature, alleviates respiratory stress, and enhances overall cow comfort [4].

Traditionally, one effective method for cooling dairy cows utilizing water is through a time-based system known as sprinklers. These sprinkler systems are designed to wet the cows’ skin with water droplets, facilitating heat removal through evaporation, especially when paired with fans. In contrast to misters, which produce fine droplets that can increase humidity, sprinkler systems deliver coarser droplets that penetrate the hair coat and reach the skin, thereby promoting more efficient heat loss. Research indicates that well-managed sprinkler and fan systems can help maintain milk production and reduce heat-stress behavior during hot conditions. On average, sprinkler cooling systems use approximately 15–20 gallons (57–76 L) of water per cow per day, with seasonal totals ranging from 500 to 1500 gallons (1890–5680 L) per cow, depending on the climate and system design (Michigan State Extension; Progressive Dairy).

In a situation where there are no cows in the cooling areas, reducing water wastage on cow cooling will reduce dairy production costs, improve the well-being of cows, and save water for future generations [5]. Furthermore, water consumption in dairy husbandry is one of the bottleneck factors for sustainable milk production [6,7]. Water is a precious resource, and reducing water wastage is a priority for sustainable agriculture [8]. A key challenge to saving water for heat abatement in cows is to predict where cows are located in the barns in order to dispense water in a strategic way and to prevent the wasting of water.

An opportunity for detection of the presence or absence of cows in the headgates of dairy barns is real-time object detection using approaches from computer vision [9]. To this end, real-time object detection using computer vision models, among which YOLO, Cascade R-CNN, and Faster R-CNN are commonly used for real-time streaming of videos from surveillance cameras [10]. The significance of computer vision applications in livestock systems has been on the rise due to their ability to produce real-time, non-invasive, and precise information at the individual animal level. Notably, various digital technologies such as wearable sensors [11], robotic milking systems [12], infrared spectrometry [13], and computer vision systems have been implemented in livestock operations to improve their efficiency and productivity [14,15]. For the purpose of covering large fields of vision in surveillance settings, panoramic or fisheye cameras are used [16]. The challenge regarding fisheye cameras is to dewarp the resulting images in order to stream them into computer vision models, because of the distortion of the images. In addition, the dewarping of fisheye camera footage can result in loss of resolution in the dewarped images. This can be a problem for computer vision applications [17].

The current study aims to reduce water consumption on farms in an innovative way by guiding sprinkler activity using artificial intelligence (AI)-enhanced detection of cows present in headgates. Specifically, the project determines the interaction between cows and their location in sections of headgates using AI-assisted location of cows in their barns. This study will analyze real-time images to determine which locations in a dairy barn should be targeted for cooling cows using customized water dispensation.

## 2. Materials and Methods

### 2.1. Dataset

The dataset for training an object detection model originated from three Axis (Lund, Sweden) fisheye cameras installed above a pen of 100 × 25 m with 400 cows at a Midwestern commercial dairy farm in the USA with 9000 Jersey–Holstein Friesian crossbred cows. The cows were housed in a cross-ventilated barn and milked in a rotary parlor 3 times per day, the average milk production was 40.8 kg per day. The video footage of 2 h long each, from the three different camera locations with 33 m between each camera at 10 m height in the ceiling of the barn, was used to train a Yolov5s model as described below. The two classes of detected headgate segments were segments empty of cows and segments occupied by cows. Every 180 min video was transformed into 100 dynamic example images throughout the day, capturing a wide array of environmental variations between late spring and the end of fall of 2023. The chopped images contained 1100 instances of cows visible in headgate segments and 600 instances of empty headgate segments.

### 2.2. Software

The software used for the workflow was Python version 3.11.6 [18] and ffmpeg version 6.1 [19]. Among the libraries used were OpenCV [20] version 4.12.0, defisheye version 1.4.1 [21], and Pandas version 2.3.0 [22].

### 2.3. Pre-Processing of Images

Each picture frame described above was dewarped using the defisheye library in Python, a Python code example for the dewarping step. For dewarping the video files, the field of view (FOV) was set to the default value of 180 degrees, while the preferred FOV (PFOV) was set to 140 degrees, as required by the focal length of the lens. Because of the large distortion of the original warped video, a PFOV of 140 degrees was the optimal choice during the dewarping process. Two hundred randomly selected images were dewarped, and they required labeling using two classes: ‘cow’ and ‘empty’ for the training of the model. For the purpose of labeling the images, the freeware called LabelImg was used to label the 200 randomly chosen .png files using bounding boxes in Yolo .txt format.

### 2.4. Training of a Yolov5s Model

Yolov5s is a class of computer vision and detection algorithms used for object detection [23]. Briefly, Yolov5 architecture is comprised of three main parts: Backbone, Neck, and Head. The Backbone is the main body of the network. For YOLOv5, the backbone is designed using the new CSP-Darknet53 structure, a modification of the Darknet architecture used in previous versions. The Neck connects the backbone and the head. In YOLOv5, SPPF and new CSP-PAN structures are utilized. The Head is responsible for generating the final output. YOLOv5 uses the YOLOv3 Head for this purpose. YOLOv5s also introduces the following minor changes: the previous focus structure was substituted with a 6 × 6 Conv2d structure and the SPP (Spatial Pyramid Pooling) layer in a neural network was replaced with SPPF (Spatial Pyramid Pooling Fusion), resulting in a processing speed improvement of over 100 percent.

The YOLOv5s model was selected because it offers one of the best trade-offs between accuracy, speed, and model size for real-time object detection on limited-computation environments. The “s” (small) variant provides fast inference and low memory usage, making it well suited for edge devices and continuous video processing while still maintaining strong detection performance in complex scenes. Its mature training pipeline, stable architecture, and broad adoption in applied computer-vision tasks—including livestock and agricultural settings—also ensure reliable deployment, rapid experimentation, and access to a large ecosystem of community support and optimization tools. When comparing different object detection models, the EfficientDet family stands out for its ability to achieve high accuracy relative to the computing power it uses. It accomplishes this using a special backbone and a method for combining features from different parts of an image. However, this comes with some trade-offs, as it can be a bit slower and requires more effort to fine-tune [24]. Recent studies have shown that the YOLO system performs better on microcomputers, such as the Jetson, by responding faster than EfficientDet. This means that when using YOLO on these devices, it takes less time to process images and identify objects, making it a more efficient choice for real-time applications [25].

On the other hand, the DETR model, which is based on transformers, offers a simpler and more streamlined approach. It does not rely on traditional methods like anchors or non-maximum suppression (NMS), which can make it easier to use in certain situations. It is particularly good at understanding complex scenes but tends to struggle with detecting small objects and needs longer training times [26]. This study considered using a Jetson microcomputer to perform detections. However, we found that the delays in processing caused by certain advanced technologies, along with the need for additional adjustments to enhance performance on smaller devices, would make deployment more challenging. As a result, we decided not to pursue this option [27]. In practical terms, as this study requires fast results and easy deployment, YOLOv5s is often the go-to option.

The Yolov5s [23] algorithm by Ultralytics (Ultralytics, Los Angeles, CA, USA) was used, and the model was trained for 1700 epochs with a batch size of 16 for the data set of 200 labeled images.

For model training, the data were partitioned into two random sets using scikit-learn [28] in Python: training images and validation images. In this scenario, 90 percent of the images were used for training the model and the remaining 10 percent were used for validation purposes. The performance metric for optimizing the gradient descent for the model training was mean average precision (mAP). An early stopping rate to identify the best performing prediction model is included in the model training procedure [29].

### 2.5. Water Consumption

The sprinklers operated on the commercial dairy farm required 2.83 L of water per minute during their operation. The sprinkler system from Seneca Dairy Systems (Seneca Falls, NY, USA) has been designed to activate water sprinklers based on ambient temperature. Specifically, at temperatures exceeding 22.2 °C, the system is programmed to run the sprinklers for one minute at 15 min intervals. At higher temperatures exceeding 32.2 °C, the system adjusts the sprinklers to run for one minute every 5 min. These settings have been put into place to ensure the optimal functionality of the sprinkler system and to maintain a healthy environment for the surrounding area.

The equation to estimate the water savings when the temperatures were exceeding 22.2 °C is explained below and shown as Equation (4).

The water savings per section (liters) is equal to the average time that the headgates sections were empty multiplied by the number of sprinklers per headgate section and by the average hours per day that the sprinkler should be on in addition to being multiplied by the number of days with the threshold temperature. In order to estimate the water savings when the temperature exceeds 32.2 °C, the same equation as above was used but a multiplier of 3 was added to the equation.

### 2.6. Validation

To evaluate the performance of the model, it was important to perform object detection using diverse inputs that were independent from the data used to train the model. For this end, during the validation process, 10 min of video captured in a different location and of a lower quality were presented to the best-fitting Yolov5s model. Two types of validation sets were presented to the model: a video in mp4 format and 100 dewarped .png images selected at random from the same video. During the validation process, three additional videos were used to calculate water usage and water savings.

Four videos were produced at a resolution of 1080 × 1080 and an ISO setting of 400, capturing various conditions of the barn, among which lightning occurred between mid-day and afternoon hours when the highest temperatures were expected. In addition, humans and trucks did not influence the training and performance of the model. The validation process incorporated at least one video from each camera to ensure a comprehensive evaluation:

Video number 1 = 10 min in length, video number 2 = 8 min in length, video number 3 = 90 min in length before the process of splitting and dewarping and 6 min after the process, and video number 4 = 105 min in length before the process of splitting and dewarping and 7 min after the process.

In order to dewarp the video number 2 and to generate a new validation video, frames were strung together for 30 s duration at 30 frames per second. The video number 3 had a duration of 90 min divided into 15 min intervals. A 1 min sample was taken every 15 min from the video number 3 which represents the same time interval during which the farm sprinklers were commonly switched on. This process of adding the 1 min time intervals of video footage as described resulted in six times 1 min videos or a total of 6 min of video footage. The video number 4 was 105 min in length and followed the same steps for chopping and adding periods of 1 min as conducted for the video number 3. The resulting video originating from the video number 4 was 7 min in length. The videos 3 and 4 were divided into 30 frames per second and dewarped using the library defisheye, mentioned previously. Finally, a video record was generated by stringing together the frames from video number 3 and for video number 4 into two separate videos using ffmpeg [30].

**Figure 1:**
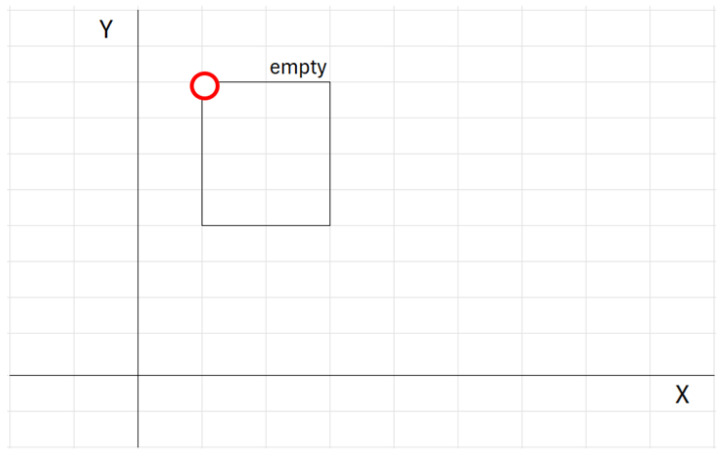
Example of x and y coordinates in the upper left hand side corner of a bounding box. The x- and y-axis represent the pixel numbers of an image.

Each of the four videos were subjected to four different object detection methods, resulting in a single list of data collected into an Excel file by Microsoft Corporation (Redmond, WA, USA (2018)). The first object detection method involved YOLOv5s detections collected into a CSV file. The second object detection method used an NVIDIA Jetson AGX Orin 64 Gb Developer Kit (NVIDIA, Santa Clara, CA, USA) for Yolov5s object detection combined with a DeepStream 6.2 tracking layer collected into a CSV file that tracked class predictions. This approach to object detection determined the duration of time during which each of the headgates was empty or occupied by cows but not the exact location in the pen. The third object detection method was a modified version of YOLOv5s that returned a CSV file with the x and y position of the top left corner of the detected bounding box (Figure 1) resulting in location of the detection in addition to class detection.

The final object detection method involved detection of classes by the human eye (fv: F. Valle) where the main investigator watched the videos and tallied the time intervals and locations for empty and occupied headgate sections into a CSV file. Cohen’s Kappa was used for quantifying the interrater agreement between the human investigator and the object detection model as described below [31]. The respective kappa value was calculated for comparing the human investigator detections of empty and occupied headgate sections with object detections from the dewarped video 2 of 8 min duration, and for the human investigator compared to 148 randomly selected images from the same video.

A full model equation for the linear regression with the outcome variable ‘*seconds*’ duration of presence or absence of cows in headgate sections was fitted according to Equation (1a,b):

Equation (1a):

(1a) seconds=intercept+b1∗video+b2∗method+b3∗video∗method+error

Equation (1b):

(1b) seconds=intercept+b1∗video+b2∗method+b3∗boxid+b4∗video∗method+error

In this context, ‘*seconds*’ refers to the average number of seconds that animals were present in each headgate section during the time intervals recorded for their presence and absence. Each time interval of the animal’s presence was measured in seconds and contributed to the calculation of the average duration of the animal’s presence in the head-gate sections. The variable ‘*method*’ represents a variable factor indicating the inference derived from the Jetson microcomputer. The x-axis and y-axis method consists in each headgate section being tracked according to the x and y coordinates of the detection in the video, as compared to the observations made by the investigator (fv, F Valle, also reference level at the equation). The error level of alpha = 5 percent was used to declare statistical significance. The only difference between Equation (1a,b) is the absence of ‘boxid’ in Equation (1a). Backward-step elimination was used to arrive at the final model Equations (2)–(4) below [32].

### 2.7. Possible Limitations

Computer vision models exhibit significant sensitivity to data quality and environmental factors. Key limitations include variations in image quality, insufficient image quantity, and inconsistencies in lighting conditions. To effectively implement the proposed approach, the data must undergo thorough validation by a knowledgeable human with expertise in AI. This study advocates for a tailored implementation strategy for each barn, as detailed in the Section 4. It is highly recommended to use a mix of different images during training to prevent any of the previously mentioned limitations. The particular rearing system employed does not influence or alter the methodology outlined in this study.

## 3. Results

A Yolov5s detection model for headgate segments occupied by cows and empty headgate segments with cows absent was generated resulting in promising prediction accuracies for dewarped images from fisheye cameras installed in the ceiling of a dairy barn.

The purpose of the AI-assisted computer vision model was to reduce excessive water usage for the cooling of cows on dairy farms using real-time AI-enhanced detection of empty space in the headgates and by locating the cows in the pens.

**Figure 2:**
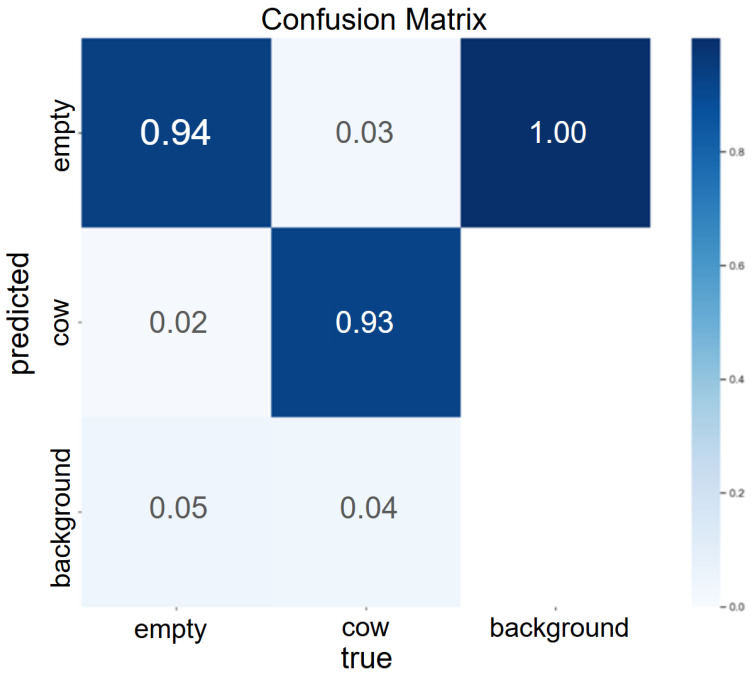
Confusion matrix generated by Yolov5s from Utralytics during the model training using 180 training images and 20 test images. The x-axis represents the true object classes, being empty and cow = occupied headgate sections in addition to the background of the image while the y-axis represents the predicted object classes with the same labels as for the x-axis; the darker blue hue represents higher prediction probabilities.

The resulting Yolov5s prediction performance after training the model for 1700 epochs had an overall mAP of 0.940. Figure 2 shows the resulting confusion matrix produced by the Yolov5s model and illustrates the agreement between observed and predicted class objects. In addition, the resulting mAP values are 0.80 and 0.73 for the 100 random images and for the 10 min dewarped video 1 using ffmpeg, respectively.

Upon assessing the model’s performance across a variety of environmental conditions, we have decided to retain the iteration utilizing 200 images for this analysis. Data augmentation and transfer learning were not utilized in this case. This decision also takes into account the potential for overfitting, as the environmental variability among the studied farm is minimal [33]. Overfitting occurs when a model learns patterns that are overly specific to the training data instead of generalizing from it, leading to poor performance on the validation set. As a result, when the model encounters unseen images, it fails to accurately make predictions or detections, indicating a lack of generalization capability.

We had detection losses of 1 to 2 headgate sections per image for the total number of detected headgate locations. In addition, we found a decreased detection accuracy after dewarping the 100 random images and 10 min video with accuracies of 0.83 and 0.54, respectively. Table 1 shows these accuracies in detail. This loss of detection accuracy for object detection using dewarped images has been reported in the literature before [34]. During the model validation step, we were able to accurately detect the presence of cows and empty headgate spaces in all four sample videos. The images dewarped from videos using the Python library known as defisheye demonstrated the adequate mean average precision (mAP) of 0.80. Among the video samples, the 8 min video 2 with higher quality performed best, exhibiting an mAP of 0.74 and lower standard deviation compared to the other videos as shown in Table 1. The fact that videos with higher resolution performed better than videos with lower resolution has also been previously reported when comparing between different qualities of pixels in videos [35]. We compared all the results of all videos with the results obtained from the 6 min video 4 for the outcome ‘minutes of duration of appearance for cows’ versus ‘empty headgate spaces’. Only the 10 min video 1 showed a significant difference in duration with empty headgates compared to the 6 min video 4, which is reasonable considering that longer videos tend to have more empty spaces compared to shorter video footage as shown in Table 1.

For the Cohen’s kappa coefficient, a measure of interrater agreement, we found 0.79 for the overall agreement between 100 images scored for empty and occupied headgate sections by the Yolov5s model and the human investigator (fv). This indicated a substantial agreement between the two raters, and it suggested that the scoring method used for the assessment tool is reliable and consistent with producing the same results across different raters [36].

We generated a final linear regression model for the outcome ‘*seconds*’, that was the duration of detection, after backward-step elimination from the full model equations, (1a) and (1b), as shown in Equation (2):

(2) seconds=intercept+video+method+video∗method+error

The linear regression analysis results for Equation (2) are shown in Table 2.

**Figure 3:**
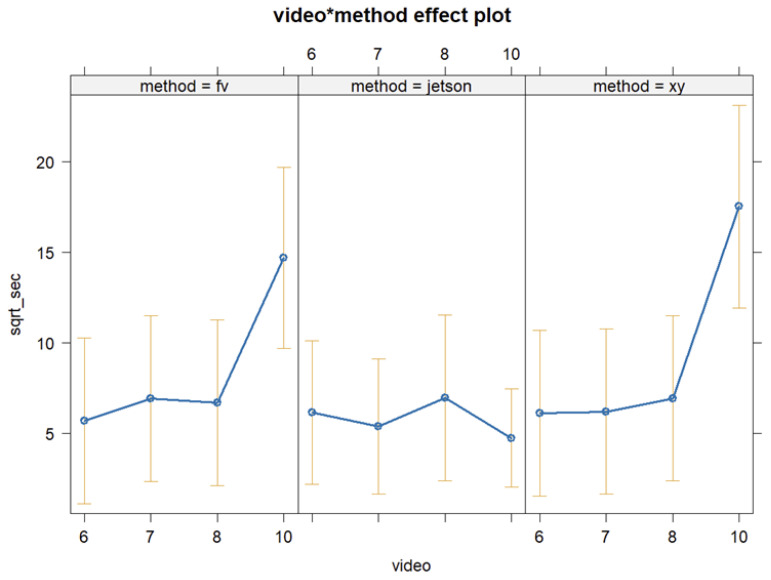
The effect plot for the final model Equation (2) for the interaction of video and object detection method being videos 6, 7, 8, and 10 on the x-axis; the y-axis represents the square root of seconds while the three panels show the results for the three methods, being a human detector (fv—F Valle), an NVIDIA Jetson AGX Orin with a Yolov5s object detection model, and ‘xy’ method tracking position using the x-axis and y-axis, representing the limit coordinates of the bounding boxes used as class label identifier; *n* = 85.

The Jetson method stands out based on the 10 min video 1, during which the detection results from the Jetson method were significantly lower than the other methods because the detection of a headgate section was at times joined across three sections as shown in Figure 3. There could be various reasons for this joining of headgate sections during the detections. One possibility is that the bounding box utilized for identifying the class ‘empty’ in the 10 min video 1 captured more than one headgate section at once. This can result in the duration of this detection as ‘empty’ to be tripled. Another possibility for the lower detection by the Jetson is that the Jetson’s tracking mechanism is designed to detect the presence of cows or empty headgate sections and to keep track of them during their presence in the camera’s field of vision. When a new bounding box replaces a previous one in the same location, the box ID changes, making it difficult to track box IDs over time. The resulting joined box IDs during object detection are the reason lead to the Jetson detection method not being entered as a predictor into the linear regression model from Equation (2) as shown in Table 3.

**Figure 4:**
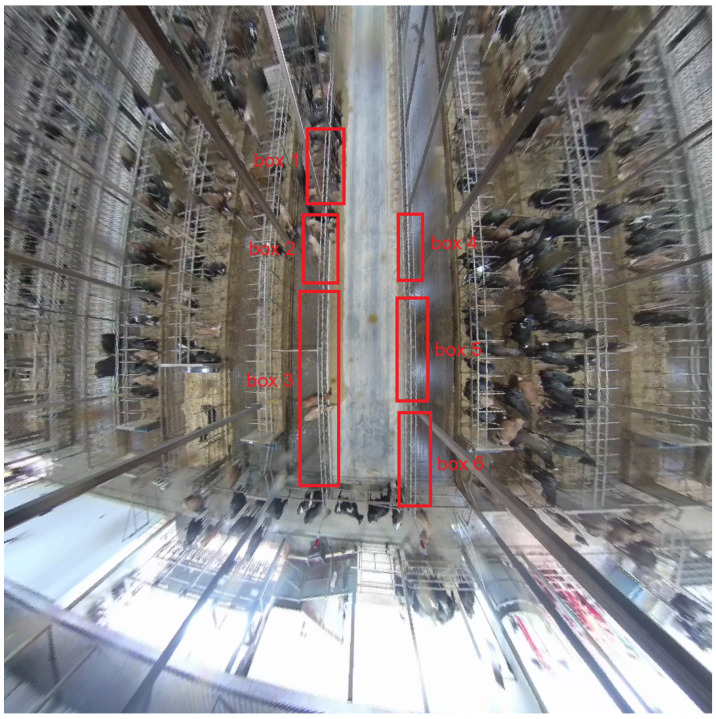
A visualization of the barn showing different boxes labeled for identification.

Our final analysis indirectly represents a study about cow behavior, where we labeled bounding boxes associated with headgate sections in six different locations of the pens as 1 through 6 (see Table 3). This analysis aimed to determine if there were significant differences in the presence or absences of cows between box 1 and boxes 3, 4, and 5, and whether cows preferred spots on the upper left-hand side of the pen or on the opposite side of the pen (see Figure 4). The preference of cows for different locations in the barn, especially under heat stress has been reported previously by Allen et al. [37].

In this context of finding locations of headgate sections more often occupied by cows, we defined a linear regression model for the outcome ‘*seconds*’ after backward-step elimination as shown in Equation (3):

(3) seconds=intercept+video+method+boxid+video∗method+error

To simplify the reporting of the results, we joined the boxes into two groups: left-side and right-side detection boxes. The left-side group included boxes 1, 2, and 3, with box 1 being located at the top left and box 3 at the bottom left. The right-side group included boxes 4, 5, and 6, with box 4 being located at the top right and box 6 at the bottom right corner of the pen. Based on the results of this study, we found that cows statistically significantly preferred spots on the left upper side of the pens.

This finding of cows preferring parts of the pen is of interest because it could result in larger locations being left without water dispensation by sprinklers and potential water savings or overheating cows in certain locations of the barn. Large areas empty of cows can be related to changes in lighting, heat stress, temperature differences in the barn, and social hierarchies of cows [38].

The final description of results concerns the water usage and potential water savings by custom steering of sprinklers. When the environmental temperature exceeds 22.2 °C, the sprinklers are set to activate water dispensation every 15 min for a duration of 1 min. In 2022, temperature registration by weather services [39] recorded 123 days with 22.2 °C, with an average of 10 h per day for the location of the dairy barn. This results in a total estimate of 4920 min for each sprinkler dispensing water at 2.83 L per minute. Considering three sprinklers per headgate section and the water usage by sprinklers being 2.83 L per minute, the total water consumption per year per section adds up to 41,904.5 L. We used an average of six headgate sections during this project, which resulted in 6 × 41,904.5 L = 251,427.05 L of water consumption per year.

Also in 2022, temperature registration by weather services recorded 7 days with 32.2 °C, with an average of 7 h per day. This results in a total estimate of 588 min for each sprinkler dispensing water at 2.83 L per minute. Considering three sprinklers per headgate section and the water usage by sprinklers being 2.83 L per minute, the total water consumption per year per section adds up to 1669.37 L. We used an average of six headgate sections during this project, which resulted in 6 × 1669.37 L = 10,016.2 L of water consumption per year.

The total water consumption for all six headgates studied during the times and duration when water was being dispensed was 261,443.25 L. To calculate the water savings, we used the datasets from the 7 min video 3 and the 6 min video 4.

The best scenario was using the average duration of empty headgate sections derived from the 7 min video, which was 1 min and 11 s (SD: 63 s) per headgate, and the worst scenario was from the 6 min video, which was 47 s (SD: 52 s) per headgate.

**Figure 5:**
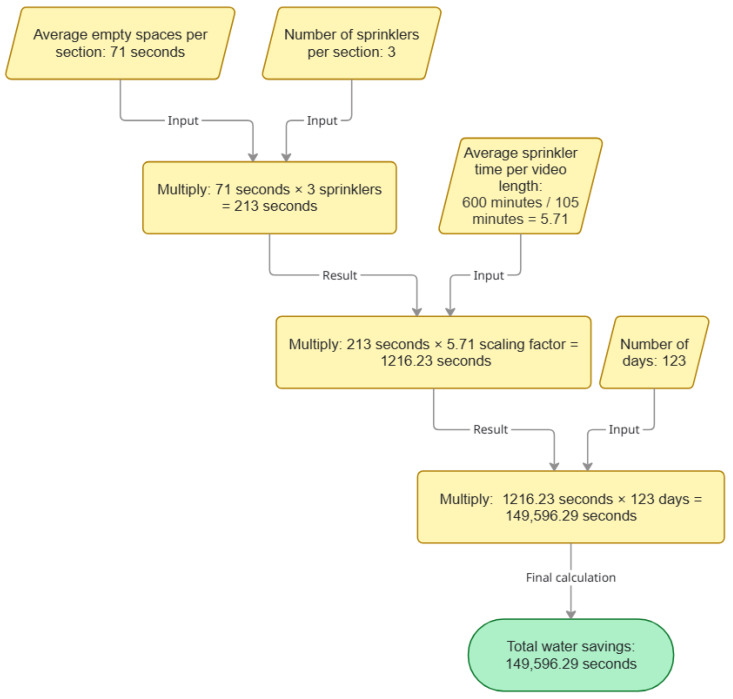
Illustration of the methodology employed for calculating water savings, utilizing Equation (4) as the foundational framework for analysis.

Since the 7 min video 3 was derived from a 105 min video, Equation (4) represents water savings when temperatures were between 22.2 °C and 32.2 °C. Figure 5 presents a comprehensive visualization of the calculations for water savings, utilizing Equation (4) as the foundational basis for analysis as follows:

(4) 71seconds∗3sprinklers∗5.71∗123days=149,596.29seconds

- 71 s = Average of empty spaces per section;
- 3 sprinklers = Considering 3 sprinklers per section;
- 5.71 = Average hours per day the sprinkler should be on was 10 h (600 min), the 7 min video came from 105 min of video, so 5.71 represents 600/105;
- 123 days = Number of days in 2022 that had temperatures above 22.2 °C.

For temperatures above 32.2 °C and considering the same steps as above, Equation (5) was

(5) 71seconds∗3sprinklers∗4∗3∗7days=17,892seconds

- 71 s = Average of empty spaces per section;
- 3 sprinklers = Considering 3 sprinklers per section;
- 4 = Average hours per day the sprinkler should be on was 7 h (420 min), the 7 min video came from a 105 min video, so 4 represents 420/105;
- 3 = When temperatures exceed 32.2 °C, the sprinkler should be on every 5 min, which means 3 times more than Equation (4);
- 7 days = Number of days in 2022 that had temperatures above 32.2 °C.

Since the 6 min video 3 was derived from a 90 min video, Equation (4) represents water savings when temperatures were between 22.2 °C and 32.2 °C:

(6) 41seconds∗3sprinklers∗6.7∗123days=101,364.3seconds

For temperatures above 32.2 °C, and considering the same steps above, Equation (5) was

(7) 41seconds∗3sprinklers∗4∗3∗7days=10,332seconds

## 4. Discussion

Upon examining Table 1, it is evident that the 10 min video 1 would result in greater savings. Given the large length of this video, we treated the length as an outlier and preferred to use to two videos 3 and 4 for calculating the water savings. In the best scenario, the savings were 2791 min of sprinkler activity per section. Considering that for this study we used six head-gate sections per video, the savings added up to 2.791 × 6 = 16,746 min or 47,391 L of water per year. In the worst-case scenario, we observed that the savings were 1861 min per section. Considering that we used six head-gate sections per video, the savings add up to 1861 × 6 = 11,166 min or 31,600 L of water per six sections per year. In this study, we revealed that the incorporation of an AI-enhanced sprinkler solution can save a significant amount of water.

As previously discussed, substantial regions devoid of cattle may be linked to variations in lightning patterns, heat stress levels, temperature differentials within the barn environment, and the established social hierarchies among bovine populations. The approach presented during this study may support guiding cows toward empty head-gates. In this case, AI helps make informed decisions about sprinkler usage and the availability of space for cow heat abatement in the barn for future use on farms to avoid any bunching behavior [40]. A study from 2010 by researchers A. Gomez and N.B. Cook found that cows typically spend a large part of their day—around 19 h or 83 percent—away from places where they eat and walk [41].

A recent study by Geoffrey E. Dahl (2023) revealed that the revolutionary SmartSoaker system could save an astonishing 80 percent more water compared to traditional methods for cooling cows [42]. With such impressive findings, calculating the savings based on the presence or absence of the animals can potentially achieve annual savings of up to 75 percent, indicating a rapid payback period for the investment, contingent upon the specific water usage metrics [43].

Furthermore, customizing the water distribution according to the location of the cows in the barn, water will be saved while heat stress is prevented at the same time. This saves resources and costs and prevents and alleviates cows from suffering from heat stress [44].

The estimated costs for hardware and the development of an AI-based sprinkler system for cooling cows on dairy farms begin with a $2000 investment in a Jetson microcomputer capable of running up to six models simultaneously. Additionally, $200 is needed for Jetson accessories. Each surveillance camera, which monitors up to 10 head-gate sections with a maximum of 12 animals each, costs $200. This setup allows for a total of 120 animals monitored per camera and up to 720 animals per Jetson microcomputer. Furthermore, for manual labor associated with implementation, it is estimated that 10 working days, totaling 80 h, will be required. Hiring a computer science student at a rate of $27 per hour for tasks such as traveling to the farm, collecting and labeling data, training the model, and implementing the system will amount to $2160. For a farm with up to 720 animals, the total costs would be around $4560. Given the manual labor involved in data collection, this approach can be easily adapted and implemented across various barn layouts and environments. Cooling of cows only happens during the warm seasons. Therefore, the period between late spring and fall was chosen for data collection only. Because computer vision models can be highly sensitive to different conditions, future research could investigate the feasibility of developing a single comprehensive model that integrates data from multiple barns.

A significant limitation is the frequent dependence on model detection alone, which oversimplifies the assessment of thermal stress. Effective cooling management should incorporate the Temperature–Humidity Index (THI), especially when cooling systems are already in operation. THI provides a more accurate representation of the physiological heat load experienced by cattle and effectively captures cumulative and diurnal stress patterns, rather than merely reflecting conditions from a single day.

In addition, one of the concerns of this study is related to possible limitations of this implementation, for example, practical deployment is further challenged by camera contamination caused by dust, insects, moisture, and manure, which degrades image quality, increases noise and occlusion, and can result in false detections or tracking errors, thereby requiring ongoing maintenance and environment-specific calibration to ensure reliable and interpretable system outputs.

Furthermore, saving water combined with increased cow welfare could improve public opinion on dairy farming. Cooled cows are the priority of the producer, while saving water for future generations is a priority for society [45].

Additionally, having access to good-quality images was essential for training the YOLO model for object detection. The validation of the model results indicated a significant loss of prediction performance when we used dewarped videos with lower resolution. For future implementations of this application, a comparative analysis of the sample images utilized in the validation process would aid in selecting the video that exhibited the best prediction performance for object detection and that satisfied all requisites for video quality [46].

In future research, it would be beneficial to measure actual water consumption in cow cooling systems by installing water meters. These measurements can then be compared to simulated assumptions under varying micro-climatic conditions, considering the THI instead of relying solely on air temperature. This approach can be further evaluated against the proposed method.

## 5. Conclusions

In conclusion, this research demonstrates the potential of computer vision models in animal sciences, particularly in enhancing animal welfare. Using this technology, we can optimize livestock cooling systems, thus alleviating stress through automated adjustments based on behavioral patterns, such as triggering sprinklers during periods of animal absence. Furthermore, the findings indicate that computer vision can significantly contribute to environmental resource conservation, achieving a potential reduction in water usage for cooling cows of up to 75 percent annually.

## Figures and Tables

**Table 1:** The comparative analysis of the object detection methods using images and different video lengths.

Variable ^1^	100 Random Images	10 min Video (SD)	8 min Video	6 min Video	7 min Video
mAP	0.8	0.73	0.74	0.69	0.74
Standard Deviation	(0.04)	(0.12)	(0.1)	(0.13)	(0.11)
Empty headgates (avg/section)—Yolo	NA	6 min 51 s	1 min 29 s	47 s	1 min 11 s
Empty headgates (avg/section)—Jetson	NA	2 min 7 s (32 s)	1 min 8 s (39 s)	1 min 13 s (55 s)	1 min (55 s)
Empty headgates (avg/section)—Yolo with xy position	NA	6 min 51 s (89 s)	1 min 29 s (81 s)	47 s (52 s)	1 min 11 s (63 s)
Empty headgates (avg/section)—Human eye	NA	6 min (328 s)	1 min 7 s (52 s)	1 min 28 s (89 s)	50 s (45 s)

^1^ The variable definitions are as follows: NA: The respective data sources are not available to calculate these outcomes. Where mAP is the mean average precision metric; the four methods being a human detector (fv—F Valle), an object detection model (Yolov5s), NVIDIA Jetson AGX Orin with a Yolov5s object detection model (Jetson), and the ‘xy’ method tracking position using the x-axis and y-axis, representing a Yolov5s model used together with the limit coordinates of the bounding boxes as class label identifier.

**Table 2:** Linear regression analysis for the outcome minutes duration of appearance for the different videos and prediction methods in the project; *n* = 85 observations.

Variable ^1^	Estimate	Std. Error	T Value	Pr (>|t|)
(Intercept)	5.698	2.289	2.489	0.015 *
7 min	1.236	3.238	0.382	0.703
8 min	1.021	3.238	0.316	0.753
10 min	8.998	3.396	2.649	0.009 **
7 min: jetson	−2.000	4.232	−0.473	0.637
8 min: jetson	−0.218	4.434	−0.049	0.960
10 min: jetson	−10.411	4.161	−2.502	0.014 *
7 min: xy	−1.135	4.579	−0.248	0.804
8 min: xy	−0.189	4.579	−0.041	0.967
10 min: xy	2.400	4.964	0.484	0.630

^1^ The variable definitions are as follows: Seconds are the average number of seconds observed being empty per headgate section. Method is a factor variable for jetson and xy (x-axis and y-axis method) compared to the human investigator, fv (F Valle), and video × method is the interaction term for the interaction of video and method. *: trends for statistically significant association at the 90% confidence level **: statistically significantly associated at the 95% confidence level.

**Table 3:** Linear regression analysis compares all four datasets with two methods fv (F Valle observation) and x and y position method, and compares box 1 with all the others, each box id represents one headgate section; *n* = 85 observations.

Variable ^1^	Estimate	Std. Error	T Value	Pr (>|t|)
(Intercept)	−0.715	3.431	−0.209	0.836
7 min	1.236	3.791	0.326	0.746
8 min	1.021	3.791	0.270	0.789
10 min	9.059	4.006	2.261	0.030
xy	0.423	3.791	0.112	0.911
Box id 2	4.034	3.426	1.177	0.247
Box id 3	9.110	3.283	2.775	0.009 **
Box id 4	10.670	3.283	3.250	0.002 **
Box id 5	7.950	3.283	2.422	0.021 *
Box id 6	6.720	3.601	1.866	0.071
7 min: xy	−1.135	5.361	−0.212	0.833
8 min: xy	−0.189	5.361	−0.035	0.972
10 min: xy	1.821	5.838	0.312	0.757

^1^ The variable definitions are as follows: Seconds are the average number of seconds observed being empty per headgate section. Method is a factor variable for jetson and x-axis and y-axis compared to the human, fv (F Valle), and video × method is the interaction term for the interaction of video and method. *: trends for statistically significant association at the 90% confidence level; **: statistically significantly associated at the 95% confidence level.

## Data Availability

The data supporting the findings of this study are not publicly available due to privacy and confidentiality agreements.
